# A Case of Gas Gangrene of the Right Foot: Clinical Presentation and Management

**DOI:** 10.7759/cureus.89545

**Published:** 2025-08-07

**Authors:** Mughilan Selvaraj, Prasanna Kumar, S. Balakrishnan, Karthikeyan Selvaraj, Jesu Pencilin Yesuvadiyan

**Affiliations:** 1 General Surgery, Sree Balaji Medical College and Hospital, Chennai, IND

**Keywords:** clostridial myonecrosis, clostridium perfringens, gas gangrene, tissue necrosis, unmanaged diabetes

## Abstract

Gas gangrene is a rare, life-threatening infection caused mainly by *Clostridium perfringens* and other *Clostridium *species, such as *C. septicum*, *C. novyi*, *C. bifermentans*, and *C. histolyticum*. Other microbial pathogens also reported to cause gas gangrene are *Staphylococcus aureus* and *Klebsiella pneumoniae*. It is fulminant and associated with high rates of morbidity and mortality, especially in patients with underlying comorbidities such as diabetes mellitus. Major underlying etiology is associated with a history of trauma or surgical wounds exposed to soil and fecal matter containing the causative microbes. This is a case of a 53-year-old man with unmanaged diabetes presenting with gas gangrene in his right lower limb. He was managed by below-knee amputation. The histopathological analysis confirmed *Clostridium perfringens* as the causative organism.

## Introduction

Gas gangrene is rapidly progressive in nature, with *Clostridium perfringens *as the common causative agent. It is a gram-negative anaerobe found in the gastrointestinal tract [1]. Gas gangrene is also known as clostridial myonecrosis, as its toxins cause extensive tissue damage and soft tissue necrosis, which is mainly caused by the production of alpha toxin and other extracellular toxins [2,3]. Alpha-toxin or CPA is a 370 amino acid zinc-containing phospholipase. This phospholipase, based on its protein sequence and antigen cross-reactivity, is related to other phospholipases of other strains of *Clostridium *species [4]. These infections progress through three stages: stage 1 is initially observed as disrupted blood flow which enables clostridial growth, stage 2 is noted as bacterial proliferation and toxin production, regulated by the VirSR system and quorum sensing in *C. perfringens*, and finally stage 3 is observed as tissue necrosis and clinical disease caused by the toxins produced [3]. The consequences are adverse in unmanaged cases of diabetic individuals [1]. This is a case report of a 53-year-old unmanaged diabetic man who presented with an ulcer for six months, skin discoloration, purulent discharge, and fever. He was later diagnosed with gas gangrene and was managed by below-knee amputation and later by above-knee amputation.

## Case presentation

**Figure 1:**
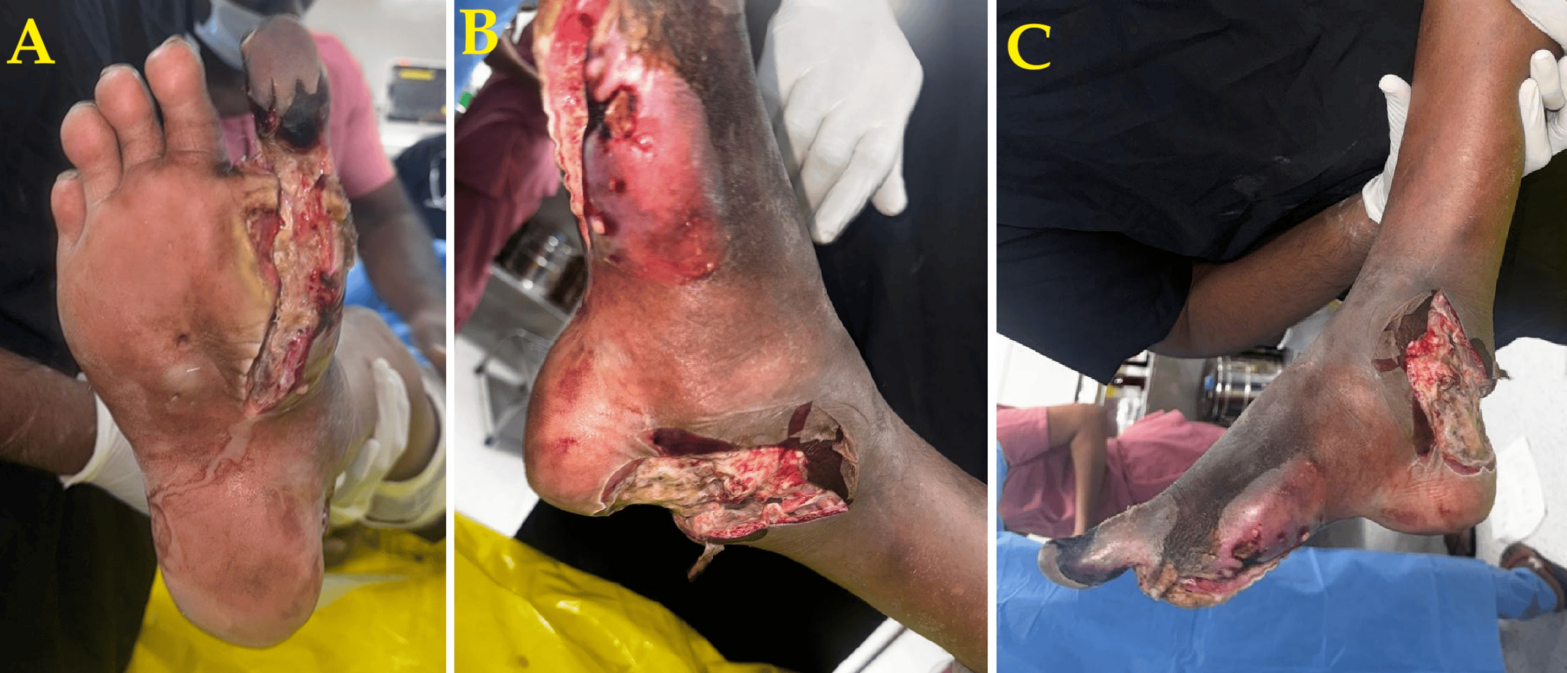
Preoperative presentation before below-knee amputation A: Dorsal view of the right foot showing a large ulcer with blackish discoloration and slough involving the first web space, suggestive of advanced soft tissue necrosis. B: Closer view of the ulcer bed with foul-smelling purulent discharge, indicating active infection and tissue breakdown. C: Surrounding skin showing violaceous hue and edema, consistent with clostridial infection spreading beyond the ulcer.

A 53-year-old man, a manual laborer by occupation, residing in a semi-urban area with limited access to primary healthcare facilities, presented with an ulcer on his right foot with an ascending infection for the last six months. It was noted as a small blister six months back, which developed into an ulcer that was debrided three days ago in another hospital. There was purulent discharge from the ulcer associated with a pricking type of pain. There was a history of pain for three days without any chills or rigor. The patient had had diabetes for eight years, which was uncontrolled and currently not managed with any medications. There was also a history of an ulcer over the plantar aspect of the left foot, which was treated. There was also a history of excision of abdominal swelling approximately eight years back. On physical examination, a 3×5 cm ulcer was noted over the first web space of the right foot. The base was slough-filled and foul-smelling and did not bleed on touch, with the surrounding skin showing early signs of necrosis (Figure 1). Peripheral pulses were absent in the posterior tibial artery on the right, while other arterial pulses were palpable. No inguinal lymphadenopathy was noted. The abdomen was soft and non-tender.

**Figure 2:**
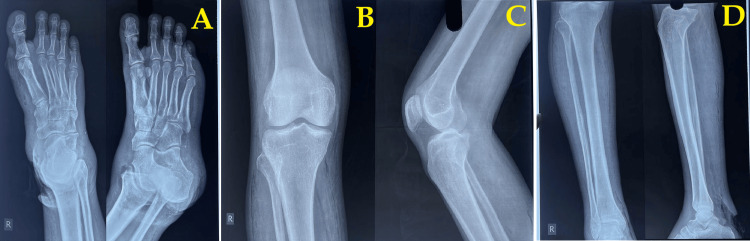
X-ray image of the infected foot A: Anteroposterior view. B: Anterior view. C: Lateral view. D: Tibia-lateral and anterior view.

Radiographic evaluation of the right lower limb revealed extensive soft tissue swelling without any clear signs of osteomyelitis (Figure 2). 

**Figure 3:**
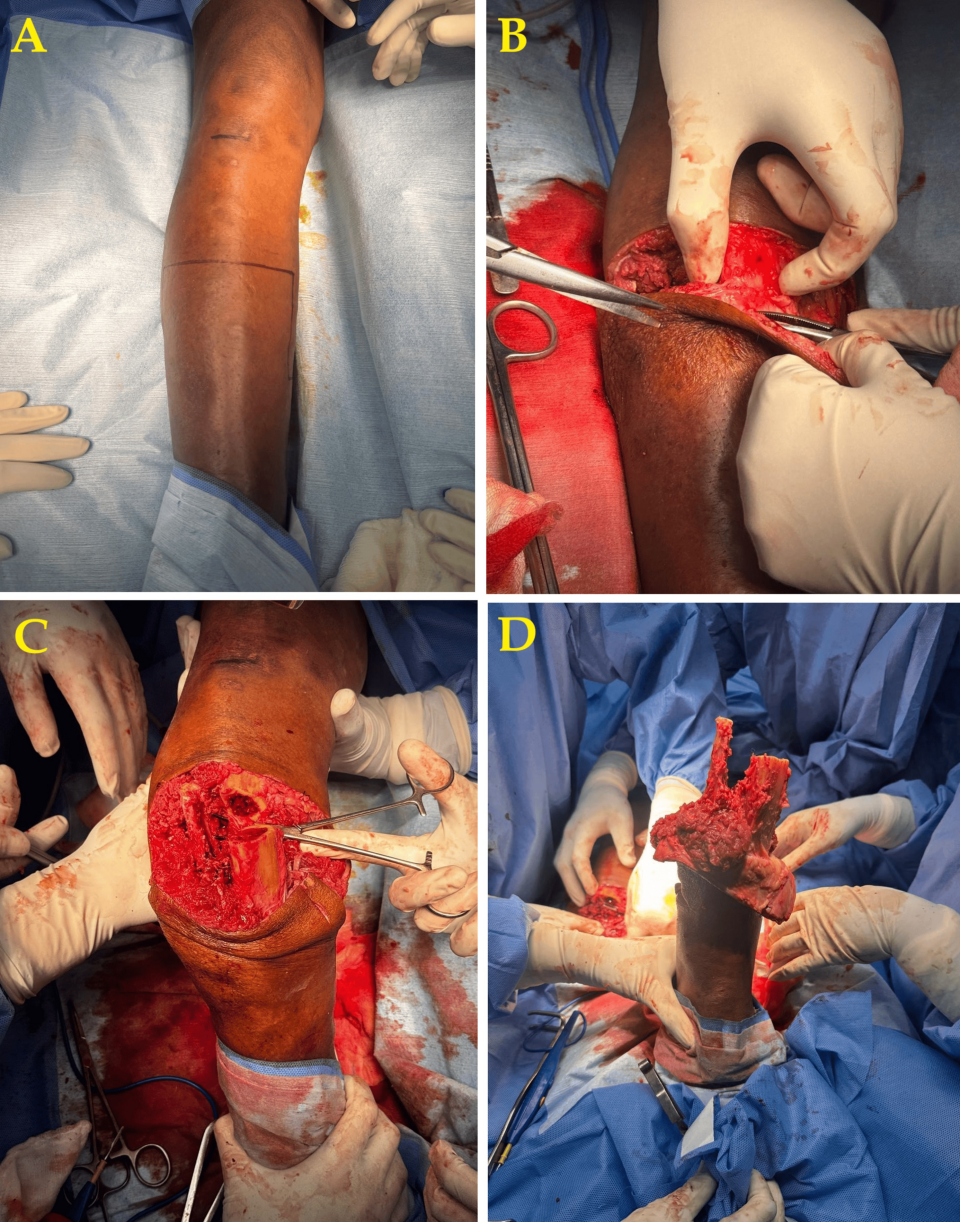
Below-knee amputation intraoperative images A: Preoperative image showing the necrotic and infected tissue. B: Intraoperative image showing the debridement of infected tissues. C: Intraoperative image showing further exploration and clearance. D: Immediate post-amputation image showing surgical closure.

A provisional diagnosis of gangrene of the right foot with a suspicion of sepsis was made. The patient was planned for wound debridement (Figure 3A-3D).

**Figure 4:**
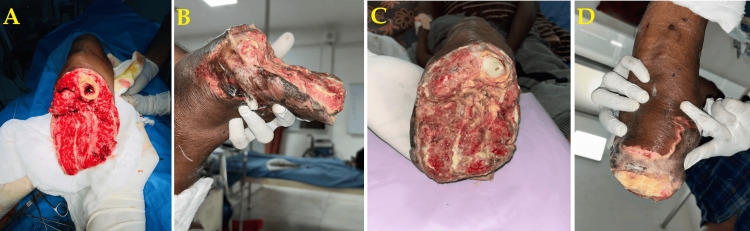
Postoperative images showing the progression of the wound (A-D)

Following the below-knee amputation, the patient was monitored closely for postoperative recovery. However, the wound demonstrated progressive signs of infection over time despite regular dressings and appropriate antibiotic therapy. Figure 4 illustrates the day-wise progression of the amputation stump, beginning with a clean closure on postoperative day 1 and gradually worsening by day 10, marked by wound gaping and purulent discharge. These clinical findings indicated a persistent, unresponsive infection.

**Figure 5:**
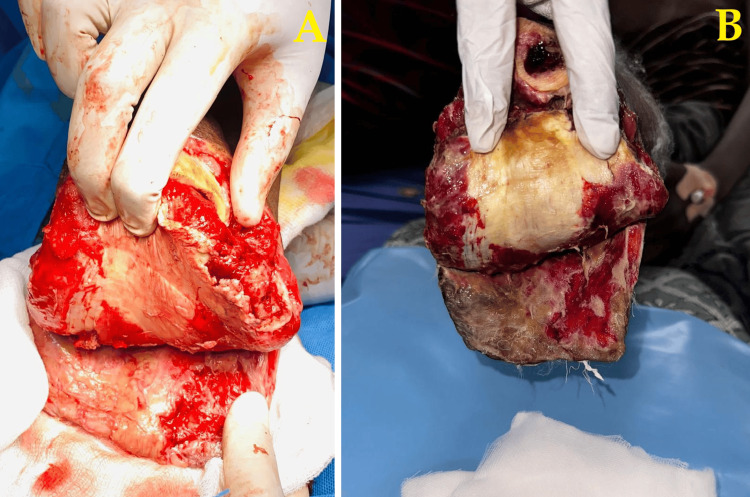
Intraoperative images during revision surgery A: Intraoperative image showing purulent discharge from the stump site during reopening. B: Exploration revealing deep-seated necrosis and unresolving infection, warranting above-knee amputation.

Given the failure of conservative wound management and the presence of necrotic tissue extending proximally, the surgical team decided to proceed with an above-knee amputation. Figure 5 captures intraoperative findings during re-exploration of the infected stump. Extensive deep tissue necrosis and unresolving infection were identified, justifying the need for higher-level amputation to control the spread and ensure patient recovery.

**Figure 6:**
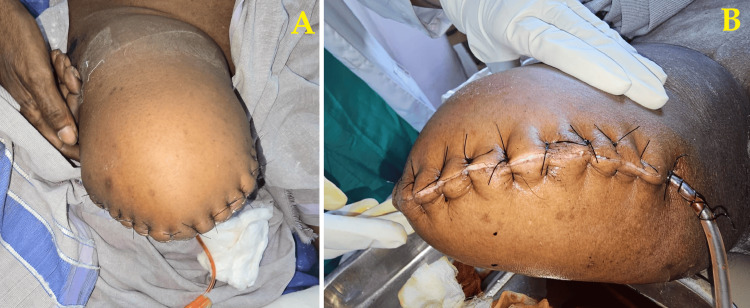
Postoperative image after above-knee amputation Final surgical stump after above-knee amputation with healthy wound margins.

The patient was managed by above-knee amputation of the lower right limb. He was managed with broad-spectrum intravenous antibiotics, initially piperacillin-tazobactam (4.5 g IV every eight hours) and metronidazole (500 mg IV every eight hours) based on local antibiogram and clinical suspicion of polymicrobial infection. However, the wound continued to deteriorate clinically, with worsening erythema and signs of systemic involvement. Given the progression and failure to respond to conservative measures, the patient was taken up for right above-knee amputation. Postoperatively, he was shifted to the surgical ward, and wound care was continued. Culture sensitivity from intraoperative samples later guided the antibiotic change to linezolid (600 mg IV every 12 hours) targeting methicillin-resistant *Staphylococcus aureus* (MRSA). Despite these measures, the infection remained unresponsive, further justifying the need for surgical amputation (Figure 6).

The patient was discharged on postoperative day 14 with no new complications. The wound swab microbial analysis confirmed the infection as clostridial myonecrosis and the causative microbe as *Clostridium perfringens*. The patient was first reviewed one week after discharge and subsequently followed up every 30 days for a total duration of six months. He was found to be recovering well physically and mentally, with no signs of any infection recurrence. 

## Discussion

Gas gangrene is a rapidly progressing and potentially fatal infection caused predominantly by *Clostridium perfringens*, although other clostridial species and bacteria like *Staphylococcus aureus* and *Klebsiella pneumoniae* have also been implicated [1]. It is often associated with uncontrolled diabetes mellitus, trauma, or surgical wounds exposed to contaminated environments [2]. The infection evolves through three stages: impaired blood flow enabling bacterial growth, proliferation with toxin production, and, finally, extensive tissue necrosis [3]. The alpha-toxin (CPA), a zinc-dependent phospholipase produced by *C. perfringens*, plays a central role in tissue destruction and disease severity [4]. The pathogenicity and virulence of *Clostridium perfringens* have been well studied, showing its ability to produce multiple extracellular toxins that damage host tissues [5].

Our patient, with long-standing unmanaged diabetes, developed gas gangrene following a minor skin injury. Immunocompromised individuals, such as diabetics, are particularly susceptible to rapid progression and systemic toxicity [6]. While most cases progress within 6-48 hours [7], our patient had a prolonged course over six months, likely due to intermittent care and poor glycemic control. Early symptoms such as localized pain, swelling, skin discoloration, and fever, as observed in this patient, should raise suspicion for gas gangrene in diabetics [8]. Although non-clostridial organisms like *Klebsiella pneumoniae* can also cause gas gangrene in such patients [9], *Clostridium perfringens *was microbiologically confirmed in this case.

Occasionally, gas gangrene may occur without a visible wound, and it can affect both limbs simultaneously [10]. In this patient, radiographs showed soft tissue gas without osteomyelitis, and clinical deterioration despite initial debridement necessitated a below-knee amputation. Similar cases have emphasized the importance of early and radical surgical intervention to prevent systemic spread [11]. Rare cases of non-clostridial gas gangrene involving unusual sites like the neck, face, or mediastinum have been documented, especially in dental or head and neck infections [12-14]. However, in our case, the infection was limited to the lower limb, progressing proximally despite appropriate antibiotics and debridement, eventually requiring above-knee amputation.

Delayed diagnosis and suboptimal initial management are major contributors to poor outcomes [15]. This case underscores the necessity of prompt recognition, aggressive surgical debridement, appropriate antimicrobial therapy, and strict glycemic control in managing gas gangrene in diabetic patients.

## Conclusions

Gas gangrene is a rare but aggressive infection that can rapidly progress to systemic toxicity and limb loss, especially in patients with uncontrolled diabetes. Early recognition, prompt surgical intervention, and targeted antibiotic therapy are critical to improving outcomes. This case highlights the importance of maintaining a high index of suspicion for gas gangrene in diabetic patients with chronic wounds and emphasizes the need for timely, aggressive management to prevent morbidity and mortality.
